# Comparison of the Agatston score acquired with photon-counting detector CT and energy-integrating detector CT: ex vivo study of cadaveric hearts

**DOI:** 10.1007/s10554-021-02494-8

**Published:** 2022-01-05

**Authors:** Susann Skoog, Lilian Henriksson, Håkan Gustafsson, Mårten Sandstedt, Sebastian Elvelind, Anders Persson

**Affiliations:** 1 Department of Radiology and Department of Health, Medicine and Caring Sciences, Linköping University, 581 85 Linköping, Sweden; 2 Center for Medical Image Science and Visualization (CMIV), Linköping University, Linköping, Sweden; 3 Department of Clinical Pathology, and Department of Biomedical and Clinical Sciences, Linköping University, Linköping, Sweden; 4 Department of Medical Radiation Physics, and Department of Health, Medicine and Caring Sciences, Linköping University, Linköping, Sweden

**Keywords:** Photon counting detector CT, CT-angiography, Heart, Arteriosclerosis, Calcium, Coronary vessels

## Abstract

The purpose of this study was to compare the correlation and agreement between AS derived from either an energy-integrating detector CT (EID-CT) or a photon-counting detector CT (PCD-CT). Reproducibility was also compared. In total, 26 calcified coronary lesions (from five cadaveric hearts) were identified for inclusion. The hearts were positioned in a chest phantom and scanned in both an EID-CT and a prototype PCD-CT. The EID-CT and PCD-CT acquisition and reconstruction parameters were matched. To evaluate the reproducibility, the phantom was manually repositioned, and an additional scan was performed using both methods. The EID-CT reconstructions were performed using the dedicated calcium score kernel Sa36. The PCD-CT reconstructions were performed with a vendor-recommended kernel (Qr36). Several monoenergetic energy levels (50–150 keV) were evaluated to find the closest match with the EID-CT scans. A semi-automatic evaluation of calcium score was performed on a post-processing multimodality workplace. The best match with Sa36 was PCD-CT Qr36 images, at a monoenergetic level of 72 keV. Statistical analyses showed excellent correlation and agreement. The correlation and agreement with regards to the Agatston score (AS) between the two methods, for each position as well as between the two positions for each method, were assessed with the Spearman´s rank correlation. The correlation coefficient, rho, was 0.98 and 0.97 respectively 0.99 and 0.98. The corresponding agreements were investigated by means of Bland–Altman plots. High correlation and agreement was observed between the AS derived from the EID-CT and a PCD-CT. Both methods also demonstrated excellent reproducibility.

**Supplementary Information:**

The online version contains supplementary material available at 10.1007/s10554-021-02494-8.

## Introduction

Energy-integrating detector computed tomography (EID-CT) is used for detection of atherosclerotic disease [1–3]. Photon counting detector CT (PCD-CT) technology has recently been introduced [4, 5], with expectation for improved clinical applications [6–9]. While EID-CTs convert incoming photons into electric currents using scintillator and photodiode layers indirectly, PCD-CTs directly convert X-ray photons into proportional electric signals using semiconductor materials. These technical characteristics of PCDs offer various advantages over conventional EID technology. Higher spatial resolution can thereby be achieved due to smaller PCD detector pixels. With the PCD technology, low-weighting of low-energy photons leads to better image contrast. This new technology, along with techniques for rendering energy-resolved data, reduces electronic noise resulting in higher dose efficiency, especially in low dose examinations. The reduced level of electronic noise not only results in less image noise but also to fewer streak artifacts and more stable Hounsfield units (HU) numbers [4, 5, 10, 11]. More energy thresholds can be applied, making advanced material decomposition possible [4]. This is a feature expected to have large clinical benefits in coronary CT angiography imaging and characterization of atherosclerotic plaques [12, 13].

In clinical practice, coronary calcifications are identified by using Agatston score (AS) evaluations [2, 12, 14–16]. AS has shown a high negative predictive value, as an AS of 0 strongly correlates with a lack of cardiovascular events over the following 5 years [12, 17]. A disadvantage of the AS is the standardized 3 mm slice thickness, leading to partial volume averaging and calcium blooming artifacts (CBA) [4, 18] which may make calcifications appear larger than their true size [16]. Also, partial volume averaging may lead to an underestimation or complete neglect of smaller and less dense calcifications. The result of these misrepresentations has been shown to lead to significant intra- and inter-scan variability for the AS [5, 10, 14, 15].

The consequence of partial volume averaging may be false negative AS of smaller and less dense calcifications. Patients with low calcium score are at higher risk compared to those with zero calcium, and medical therapy might be considered [13, 16, 19].

In a study using an anthropomorphic phantom, Van der Werf et al. reported comparable CAC scores for routine clinical protocols between conventional CTs and PCD-CTs. Furthermore, they showed PCD-CT to have increased detectability and accuracy in CAC volume estimation at reduced slice thickness [20]. Symons et al.´s study demonstrated the potential of PCD technology to improve CAC score image quality and/or reduced radiation dose while maintaining diagnostic image quality. Their study was performed with a cardiac CT phantom, ex vivo hearts and asymptomatic volunteers [21]. Both studies were performed with a lower and a higher threshold setting in the PCD-CT and not with different monoenergetic levels. Eberhard et al. investigated CAC score in PCD-CT compared to EID-CT with different doses of radiation, different QIR and different monoenergetic levels. The study showed decreasing CAC scores at increasing QIR levels and increasing keV levels [21].

The purpose of our study was to compare the correlation and agreement between the AS derived from an energy-integrating detector CT (EID-CT) and an photon-counting detector CT (PCD-CT). Reproducibility was also compared.

## Material and methods

### Ethics

The study was approved by the Swedish Ethical Review Authority (Dnr 2020-06114.).

### EID-CT and PCD-CT image acquisition and reconstructions

Five cadaveric hearts were positioned in a chest phantom (N1 Lungman; Kyoto Kagaku Co. Ltd, Japan) and scanned in both an EID-CT (SOMATOM Force; Siemens Healthineers, Forchheim, Germany) as well as in a PCD-CT prototype (SOMATOM Count Plus; Siemens Healthineers, Forchheim Germany*)*.

ECG-gating was not available in the PCD-CT. The vendor-provided spiral cardiac CAC Score protocol on the EID-CT is ECG-gated and ECG dose modulated. Thus, the expressed CTDI_vol_ is based on the average tube current of the whole scan including both low and full dose cardiac phases. As only the full dose phases were used for image reconstructions, the expressed CTDI_vol_ on the EID-CT, would render a too low radiation output on the PCD-CT. In order to get non-gated spiral protocols with an equal radiation output on both systems, we therefore performed the following procedure on the chest phantom before the first examination:

1. A CAC Score ECG-gated spiral scan of the phantom was made using a synthetic ECG on the EID-CT. Automatic exposure control (CARE Dose4D, Siemens Healthineers), vendor recommended Q. ref. mAs of 80 and ECG dose modulation was used. Image reconstructions were made during the full dose phase at 70% of the cardiac cycle using a 160 mm FoV. The dedicated Calcium Score kernel Sa36, as well as a 3 mm slice thickness with 1.5 mm increment were applied, as recommended by the vendor.
2. In total, nine non-ECG-gated spiral test scans were made on the EID-CT with automatic mAs exposure control (CARE Dose4D, Siemens Healthineers) using different Q. ref. mAs settings between 10 and 50. All scans were reconstructed in the same manner as the ECG-gated spiral.
3. The noise level in each test scan was determined by the placement of equal sized regions of interest (ROI) in the slices with the same slice position and at the same location in the image. By comparing the standard deviation (SD) in the non-ECG-gated scans with the SD in the ECG-gated scan a suitable Q. ref. mAs setting was found, i.e. the one rendering equal image noise (35 mAs). This Q. ref. mAs setting was then applied in the non-ECG-triggered thorax protocol used for all the following cadaveric heart scans at the EID-CT within the study.

Scans of the cadaveric hearts at the PCD-CT were made directly after the scans on the EID-CT. By matching the CTDI_vol_ between the scans as closely as possible (CTDI_vol_ varying between 0.85 and 1.14 mGy between the different cadaveric hearts) a similar radiation output was ensured. All scans within the study were performed with a spiral protocol, using a tube potential of 120 kV. In order to evaluate the reproducibility on both systems, the phantom was scanned once and then manually repositioned, after which it was scanned again.

Reconstructions were performed using quantitative kernels for calcium scoring, i.e. Sa36 for the EID-CT data and Qr36 kernel for the PCD-CT data. Images from both systems were reconstructed with a slice thickness of 3 mm and an increment of 1.5 mm. Details on the acquisition and reconstruction parameters are summarized in Table 1.

**Table 1:** CT acquisition and reconstruction parameters for energy integrating detector CT (EID-CT) and photon counting detector CT (PCD-CT) scans

	EID-CT	PCD-CT
CT acquisition and reconstruction parameters	*(SOMATOM Force; Siemens Healthineers)*	*(SOMATOM Count Plus; Siemens Healthineers*)
Scan mode	Non-gated Spiral	Non-gated Spiral
CTDIvol (mGy)	0.88 to 1.14	0.92 to 1.12
Tube potential (kV)	120	120
Pitch	1.2	1.2
Collimation (mm)	192 × 0.6	144 × 0.4
Rotation time (s)	0.25	0.33
Monoenergetic levels (keV)		50, 65, 68, 70, 72, 150
Reconstruction technique	WFBP (standard)	IR1 (WFBP was not selectable)
Kernel	Sa36	Qr36
Slice thickness (mm)	3	3
Increment (mm)	1.5	1.5
Reconstruction field of view (mm)	160	160
Image matrix size	512	512

### Coronary calcification inclusions

A total of 26 well-defined calcified coronary calcifications with volumes between 1 and 210 mm^3^ were identified and included in the study. Four to eight calcifications per heart were analysed. The calcifications were located in the left anterior descending artery, circumflex artery and/or the right coronary artery.

### Determination and comparison of AS

The image analyses were performed by a thoracic radiologist with twenty years of radiologic experience, and approximately ten years of experience in cardiac imaging. For intra-observer reproducibility the lesions in position 1, in Sa36 and Qr36, were measured twice by the same thoracic radiologist, with more than a month between the measurement occasions. All monoenergetic levels were measured twice in the scan from the PCD-CT. For further analyses only the measurements from level 72 keV was used. (See attachment for AS in all lesions in Sa36 and Qr36. Qr36 in different monoenergetic levels. Position 1 was measured twice.)

Evaluations of the AS were performed using the semi-automatic calcium score analysis software on a post-processing multimodality workplace (Leonardo MMWP, Siemens, Germany).

**Fig. 1:**
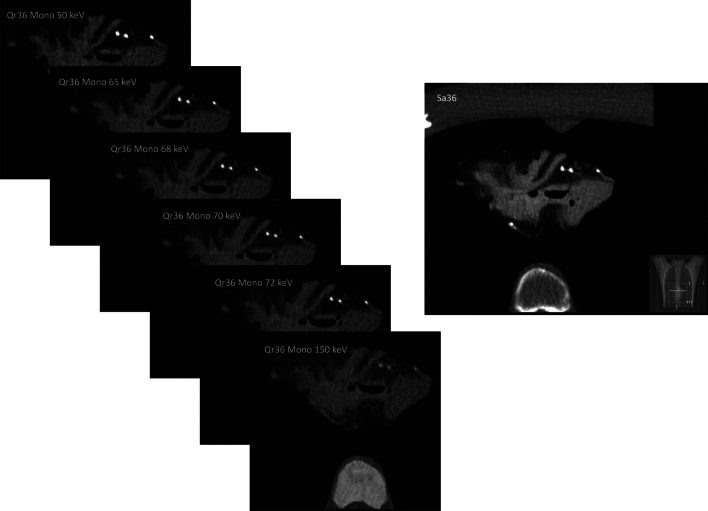
Images of one of the cadaveric hearts examined in the chest phantom (N1 Lungman; Kyoto Kagaku Co. Ltd, Japan). To the left images from the PCD-CT prototype (SOMATOM Count Plus; Siemens Healthineers, Forchheim Germany) reconstructed in Qr36 and different monoenergetic levels. To the right image from the EID-CT (SOMATOM Force; Siemens Healthineers, Forchheim, Germany) and reconstructed in Sa36

The AS values of all the included 26 calcifications were compared between the Sa36 reconstructions from the EID-CT and the monoenergetic Qr36 reconstructions from the PCD-CT. The monoenergetic levels available ranged between 45 and 150 keV. (Fig. 1).

### Image noise measurements

Image noise was defined as the SD of the mean HU value in a 1 cm^2^ ROI, measured in soft tissue/myocardium in the cadaveric hearts. Two ROIs were placed in each cadaveric heart in both positions in Sa36 (EID-CT) and Qr36, energy level 72 keV (PCD-CT).

### Statistics

Continuous data are presented as mean ± SD if normally distributed, or as median and interquartile rang (IQR) if non-normally distributed. The normality assumption was checked visually using p–p plots.

The correlation and agreement with regard to the AS, both between the two methods for each position, and between the two positions for each method, were assessed with Spearman’s rank correlation coefficient, as appropriate for non-parametric data. The agreement was investigated by means of Bland–Altman plots. The correlation and agreement regarding AS in an intra-observer analysis was also assessed with Spearman’s rank correlation coefficient and Bland–Altman plots. Although the measurements in themselves were not normally distributed, visual assessments of p–p plots found the normality assumptions for Bland–Altman plots (differences) to hold. Statistical analyses were performed using SPSS Statistics 27 (IBM, Armonk, New York). P values below 0.05 were considered statistically significant.

## Results

The best possible match for, Sa36 in the EID-CT images was Qr36, at a monoenergetic level of 72 keV in the PCD-CT images, (Table 2).

**Table 2:** Medians in EID-CT (Sa36) in respect to different monoenergetic levels in PCD-CT in positions 1(a) and 2(b)

	Sa36	Qr36mono50	Qr36mono65	Qr36mono68	Qr36mono70	Qr36mono72	Qr36mono150
N	26	26	26	26	26	26	26
25th percentile	6.800	29.200	15.325	10.800	9.525	8.950	0.100
50th percentile (median)	19.500	54.600	30.550	23.900	23.450	22.550	4.100
75th percentile	38.500	116.075	56.225	53.425	50.750	49.700	11.325

	Sa36	Qr36mono50	Qr36mono65	Qr36mono68	Qr36mono70	Qr36mono72	Qr36mono150
N	26	26	26	26	26	26	26
25th percentile	7.800	31.150	14.250	11.925	10.575	11.025	0.300
50th percentile (median)	18.850	54.300	33.200	25.000	22.950	21.500	2.950
75th percentile	35.750	109.125	64.525	52.050	59.725	48.325	14.025

**Fig. 2:**
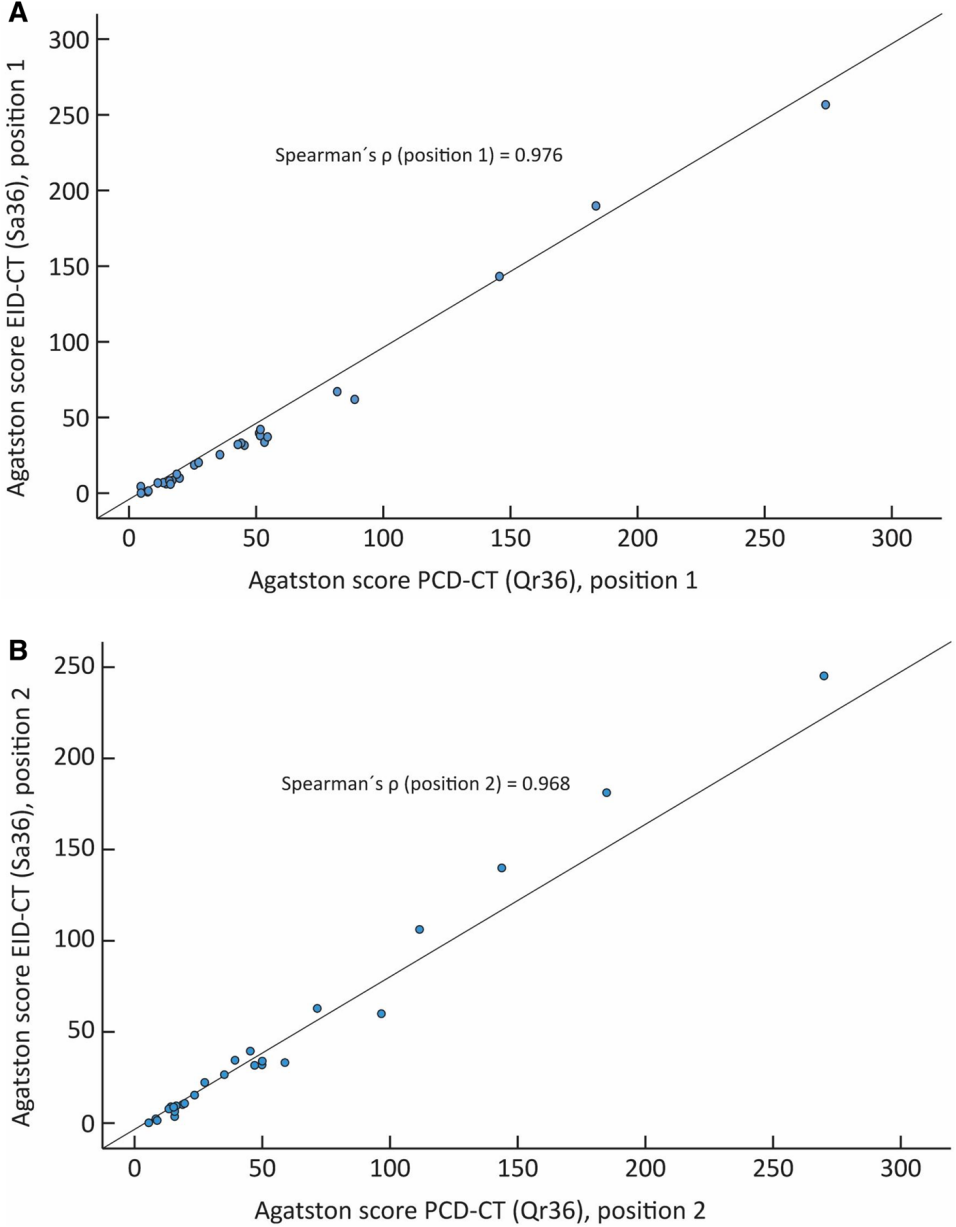
Scatter plot depicting the Agatston score correlation between the EID-CT and PCD-CT, expressed as Spearman rank correlation coefficint (**ρ**). **A** Position one: **ρ** = 0.976. B Position two: **ρ** = 0.968

**Fig. 3:**
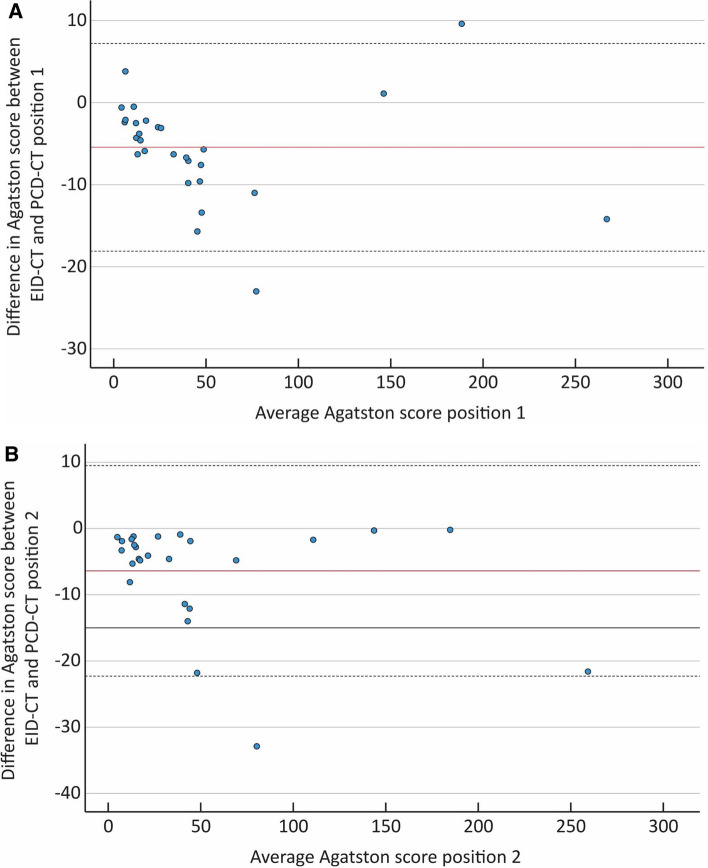
Bland–Altman plot for position 1 (**A**). The solid red line shows the mean difference between the energy integrating detector CT (EID-CT) and the Photon counting detector CT (PCD-CT) (− 5.43).Dashed lines indicate ± 2 SD (7.2 to − 18.1). Bland–Altman plot for position 2 (**B**). The solid red line shows mean difference between the EID-CT and the PCD-CT (− 6.39). Dashed lines indicate ± 2 SD (9.5 to − 22.3)

The correlation between the PCD-CT and EID-CT for position one and two with regards to the AS was analysed with Spearman’s rank correlation coefficient and showed ***ρ*** = 0.98 and 0.97, respectively (p < 0.001) (Fig. 2). The Bland Altman mean difference and 1.96 standard deviations (SD) upper and lower limits of agreements for the AS between the EID-CT and PCD-CT were − 5.43 (7.2 to − 18.1) and − 6.39 (9.5 to − 22.3) for position one and two, respectively, (Fig. 3).

**Fig. 4:**
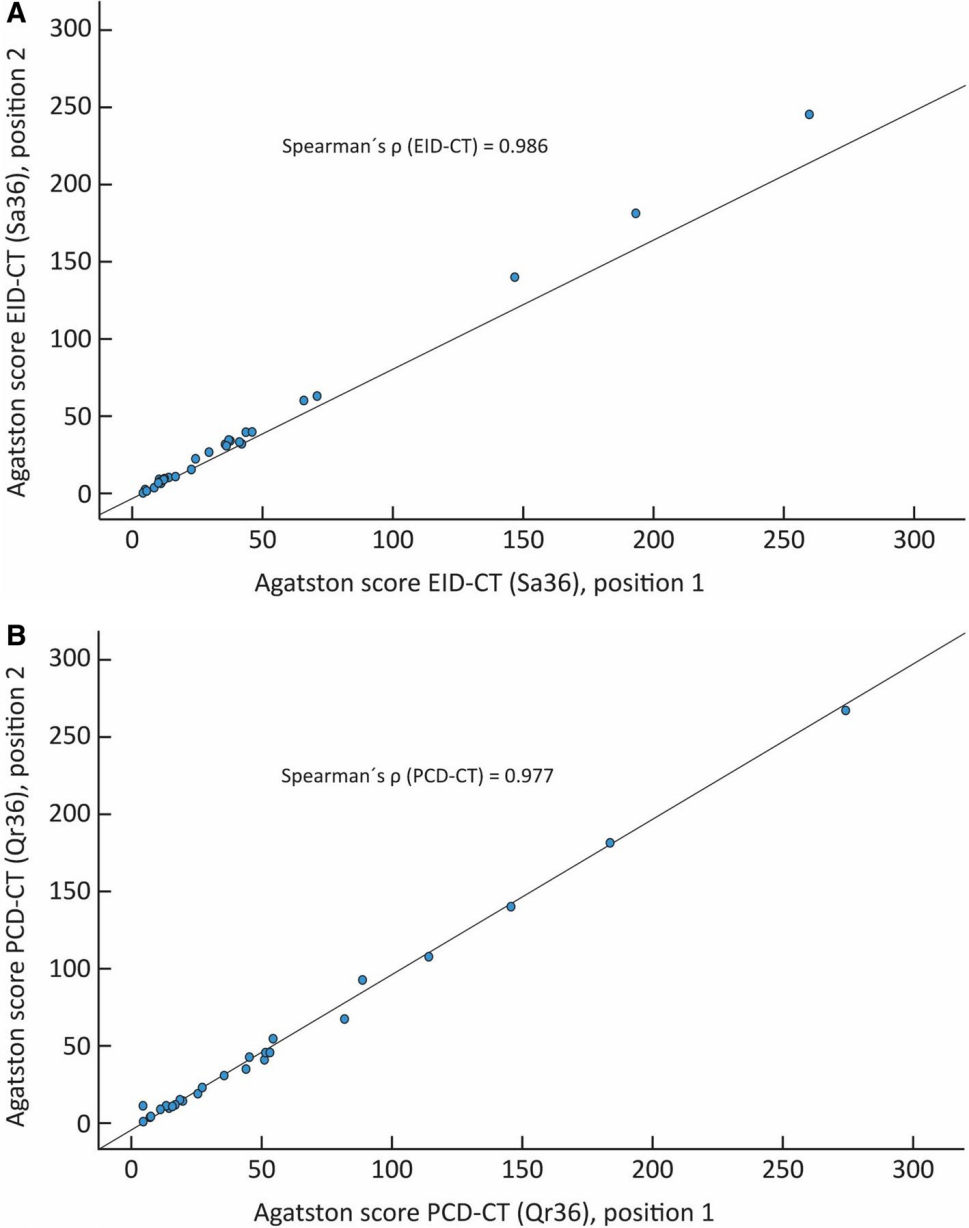
Scatter plot depicting the Agatston score correlation between position one and two in the EID-CT and PCD-CT, expressed as Spearman rank correlation coefficient (**ρ**). **A** EID-CT: **ρ** = 0.986. **B** PCD-CT: **ρ** = 0.977

**Fig. 5:**
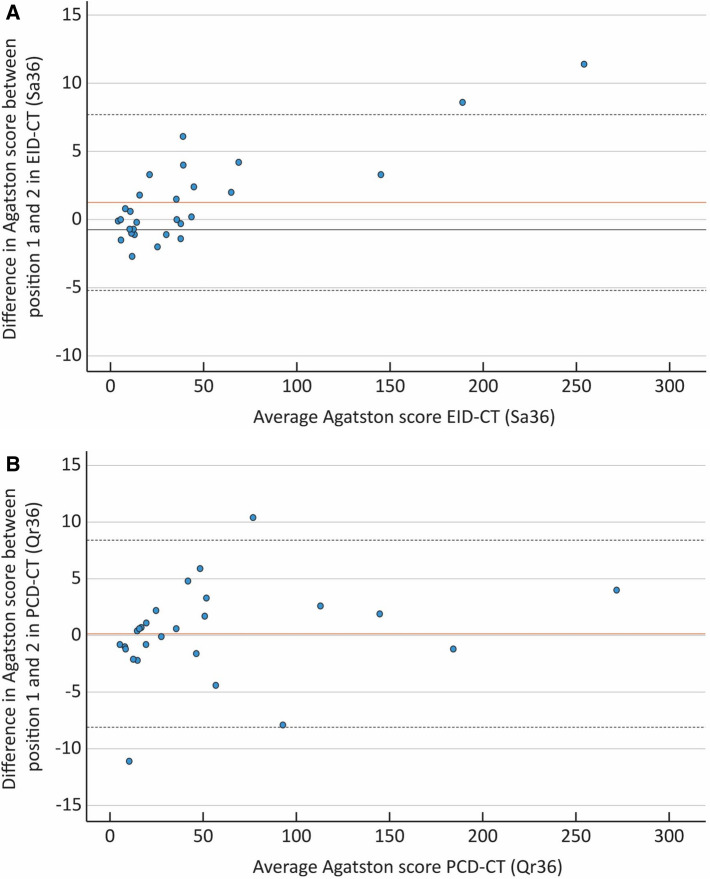
Bland–Altman plot for the energy integrating detector CT (EID-CT) (Sa36) (**A**). The solid red line shows the mean difference (1.26) and the dashed lines indicate ± 2 SD (7.7 to − 5.2). Bland–Altman plot for the photon counting detector CT PCD-CT (Qr36) (**B**). The solid red line shows mean difference (0.14) and the dashed lines indicate ± 2 SD (8.4 to − 8.1)

The correlation between position one and two for the EID-CT and PCD-CT with regards to the AS was (***ρ***) = 0.99 and 0.98 (p < 0.001) respectively (Fig. 4). The Bland Altman mean difference and 1.96 SD upper and lower limits of agreements for the AS between position one and two were 1.26 (7.7 to − 5.2) for the EID-CT and 0.14 (8.4 to − 8.1) for the PCD-CT, respectively. (Fig. 5).

**Fig. 6:**
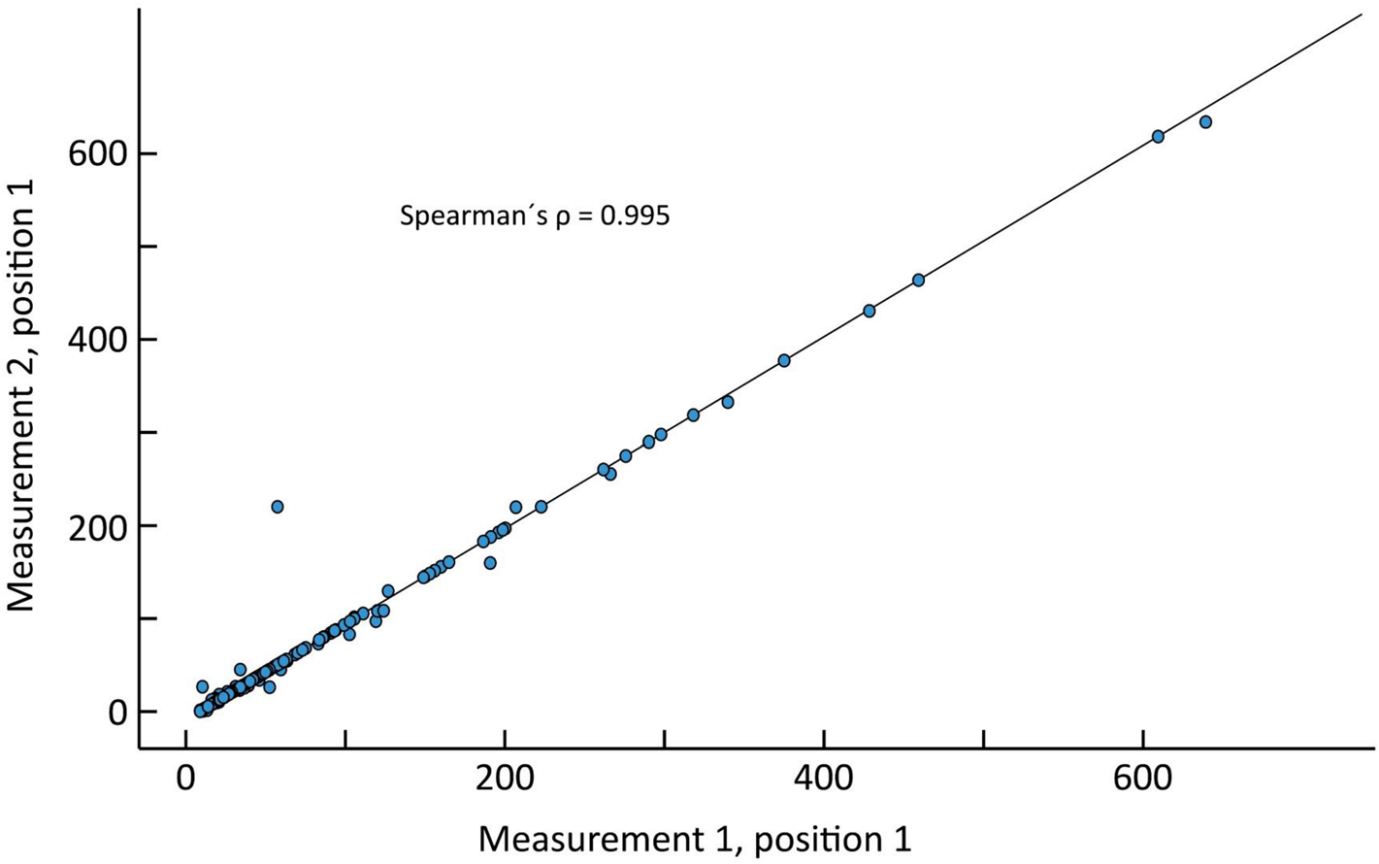
Scatter plot depicting the Agatston score correlation between two measurement occasions of position one, expressed as Spearman rank correlation coefficient (**ρ**) = 0.995

The correlation between the two measurement occasions in position 1 with regards of AS showed a (***ρ***) = 1.00 (p < 0.001), (Fig. 6).

**Fig. 7:**
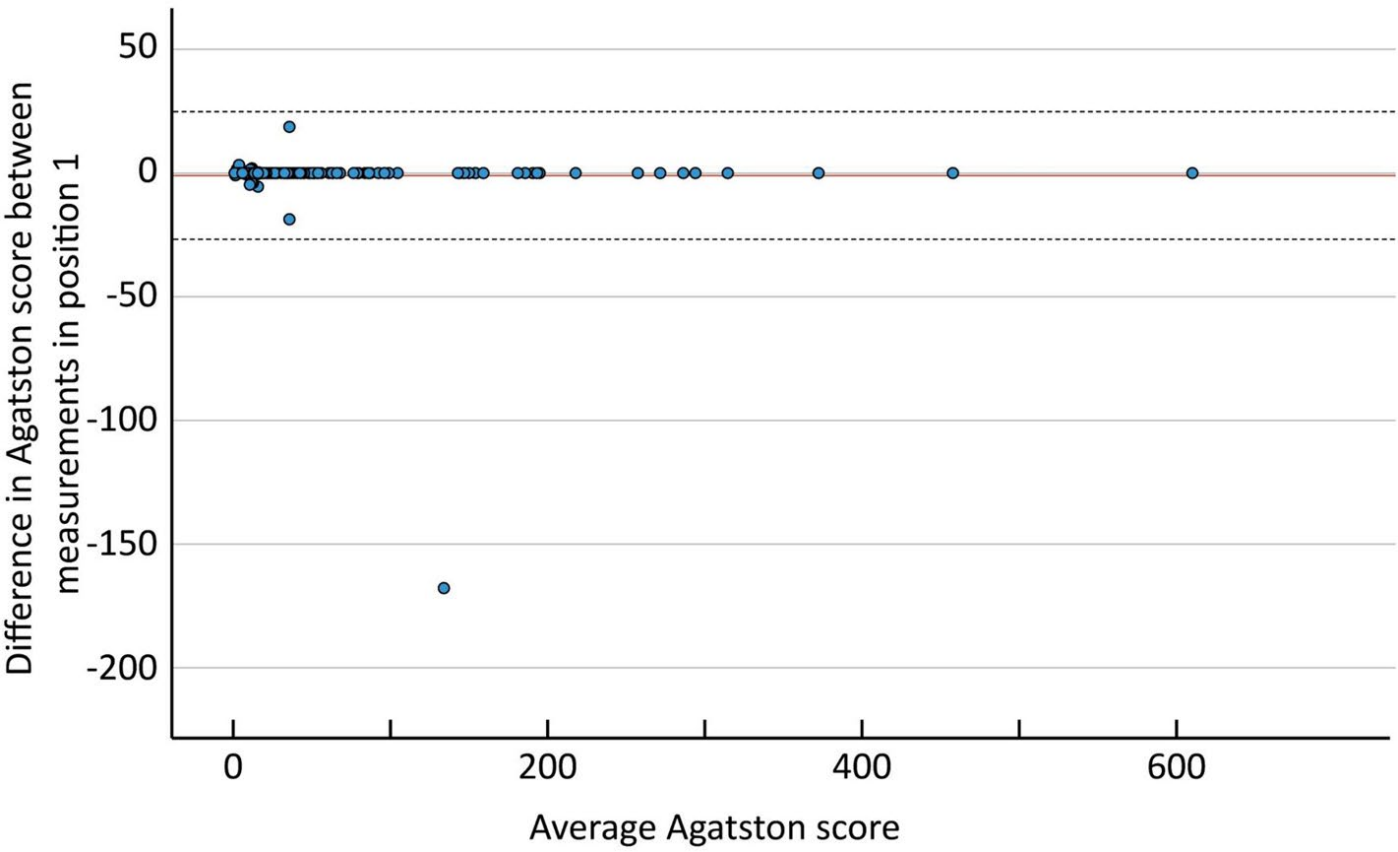
The Bland–Altman plot for position 1. The solid red line shows the mean difference between the two measurements (− 1.02). Dashed lines indicate ± 2 SD (24.8 to − 26.8)

The Bland Altman mean difference and 1.96 SD upper and lower limits of agreements for the AS between the two measurements in position one was − 1.02 (24.8 to − 26.8). (Fig. 7).

The average image noise in the EID-CT Sa36 and the PCD-CT Qr36 monoenergetic (72 keV) was 12.2 (± 2.1) and 14 (± 2.1) HU, respectively.

## Discussion

In this ex vivo study of cadaveric hearts, there was an excellent correlation and agreement between the AS derived from an EID-CT and a PCD-CT Also, both methods demonstrated an excellent reproducibility.

Measurement of the AS has long been the clinical standard for quantification of coronary calcium and still remains the most commonly used CAC score in clinical practice [12, 15, 16]. The PCD-CT is a promising technique on the verge of becoming clinically feasible. When introducing a new technique for clinical examination, it is important to determine if well-established scoring methods, such as the AS, remain reliable for early detection and risk stratification of CAD.

The augmented PCD-CT detector technology, counting every incoming photon, resulted in a slight AS overestimation tendency according to the Bland Altman analysis. In PCD-CT, calcification attenuation values acquired at 120 kV are higher than those measured in EID-CT scans. This is due to improved weighting of low-energy photons. To adjust for this as much as possible, monoenergetic images reconstructed at the keV level rendering similar HU values as those in 120 kV images should be used. We investigated the vendor-provided monoenergetic levels at 50, 65, 68, 70, 72 and 150 keV. The best possible match turned out to be reconstructions at 72 keV. If further keV levels were added in the gap between 72 and 150 keV, the slight tendency toward overestimation using the PCD-CT may potentially be compensated for, likely resulting in further improved correlation.

The historical tie between the AS and 3 mm slices, limited the improvements possible with the spatial resolution provided by the PCD-CT technique in this study.

Both the EID-CT and the PCD-CT exhibited good reproducibility which, at least to some extent, may be explained by the average noise being similar between the methods.

The intra-observer reproducibility was excellent. There was one outlier, due to incorrect measurement the first time, in the stack with keV level 65. However this stack was not used in further analyses. In the other analyses we used Qr36, monoenergetic level 72 keV (PCD-CT) and Sa36 (EID-CT).

PCD-CT technology may have additional benefits for CAC scoring, which were beyond the scope of this study. For instance, improved quantification of low or intermediate CAC scores and better evaluation of the distribution and shape of calcifications. This could lead to the method having an even higher prognostic value and better reproducibility than the current AS [5, 20, 22]. In addition, the improved HU stability, and the lower degree of electronic noise of PCD-CT, may lead to a more reliable CAC score at a lower radiation dose and detection of smaller calcified coronary lesions [21]. Further studies evaluating the possibilities for improved segmentation and quantification of coronary calcifications with the thinner slice thickness possible with PCD technology would be of interest.

The data in our study correspond with results in prior studies aiming to compare CAC scoring in PCD-CT to EID-CT for clinical routine protocols. Werf et al. also showed PCD-CT to be superior for detection of CAC at reduced slice thickness which provided more accurate volume scores [20]. R. Symons et al.´s study demonstrated the potential of PCD technology to improve CAC score image quality and/or reduce radiation dose while maintaining diagnostic image quality [23]. Eberhard et al.`s work suggested accurate CAC scoring using monoenergetic reconstructions, as well as a decreased CAC score with increasing strength levels of QIR and increasing monoenergetic levels [21].

There are some limitations in our study. Results have been generated with a PCD-CT prototype scanner not yet approved for routine clinical use, without availability of any ECG-gated scan protocols. We thereby used non-gated spiral protocols in the PCD-CT as well as in the EID-CT. This led to limited post-processing possibilities as clinical workstations are incompatible with non-ECG triggered CAC scans. Another limitation caused by the post-processing restrictions was, as mentioned above, predetermined keV levels.

Since the study used ex vivo cadaveric hearts there was no motion artifacts, and a phantom does not completely simulate an actual human. We used WFBP reconstructions at EID-CT and IR1 at PCD-CT (available IR setting was IR1-5). At the same dose level, we had expected the PCD-CT images to be less noisy than the EID-CT images. There are several potential sources that can cause this rather small difference. For instance, the relatively small ROI, the chosen keV and the difference in data processing. Only intra-reader analysis was performed. The total AS in each cadaveric heart was not measured, as two of the hearts contained calcifications in other locations such as valves and stents, which were difficult to separate from coronary calcifications.

## Conclusion

The study indicates good potential for a conversion of the established Agatston score from EID-CT to the forthcoming PCD-CT technology.

An excellent correlation and agreement was demonstrated between the AS derived from an EID-CT and a PCD-CT. The augmented PCD-CT detector technology, counting every incoming photon, resulted in a slight AS overestimation tendency. Our study showed inter-scan reproducibility to be good both in PCD-CT and EID-CT respectively.

### Supplementary Information

Below is the link to the electronic supplementary material.

Supplementary file1 (DOCX 18 kb)

## Data Availability

Data used for this study is available through the corresponding author.

## Abbreviations

AS: Agatston score
CAC: Coronary artery calcification
CBA: Calcium blooming artifacts
CT: Computed tomography
EID-CT: Energy-integrating detector CT
HU: Hounsfield units
IR: Iterative reconstruction
PCD-CT: Photon-counting detector CT
SD: Standard deviation
WFBP: Weighted filtered back projection
