# Supplementary material for: Comparison of the Agatston score acquired with photon-counting detector CT and energy-integrating detector CT: ex vivo study of cadaveric hearts
Source: Int J Cardiovasc Imaging. 2022 Jan 5;38(5):1145–55. doi: 10.1007/s10554-021-02494-8 (PMC11142966; doi:10.1007/s10554-021-02494-8)
Supplement: Supplementary file 1 — Supplementary file1 (DOCX 18 kb) [file 10554_2021_2494_MOESM1_ESM.docx]

| Plaque | **Sa36** | Qr36, mono50 | Qr36, mono65 | Qr36, mono68 | Qr36, mono70 | **Qr36, mono72** | Qr36, mono150 |
| --- | --- | --- | --- | --- | --- | --- | --- |
| 1 Pos1 | **39.7 (39.7)** | 62.5 (62.5) | 50.9 (50.9) | 49.7 (49.7) | 48.1 (48.1) | **47.3 (47.3)** | 17.6(17.6) |
| 1 Pos2 | **39.5** | 55.9 | 45.5 | 43.8 | 43.1 | **41.4** | 19.3 |
| 2 Pos1 | **67.1 (67.1)** | 103.7(103.7) | 86.3 (86.3) | 83.1 (83.1) | 78.9 (78.9) | **78.1 (78.1)** | 36.3(36.3) |
| 2 Pos2 | **62.9** | 96.2 | 75.7 | 71.7 | 69.1 | **67.7** | 22.1 |
| 3 Pos1 | **38.1 (38.1)** | 71.8 (71.8) | 54.9 (54.9) | 52.9 (52.9) | 48.7 (48.7) | **47.7 (47.7)** | 11.3(11.3) |
| 3 Pos2 | **32.0** | 73.9 | 52.0 | 49.7 | 48.1 | **46.0** | 8.9 |
| 4 Pos1 | **10.0 (10.0)** | 27.4 (27.4) | 20.9 (20.9) | 17.1 (17.1) | 16.5 (16.5) | **15.9 (15.9)** | 1.7 (1.7) |
| 4 Pos2 | **10.2** | 29.2 | 17.5 | 16.6 | 15.6 | **14.8** | 0.7 |
| 5 Pos1 | **18.7 (18.7)** | 34.9 (34.9) | 23.7 (23.7) | 22.6 (22.6) | 22.5 (22.5) | **21.7 (21.7)** | 3.2 (3.2) |
| 5 Pos2 | **15.4** | 38.5 | 25.1 | 23.2 | 20.7 | **19.5** | 2.3 |
| 6 Pos1 | **6.3 (6.3)** | 25.5 (44.2) | 15.4 (15.1) | 12.0 (10.1) | 11.3 (9.7) | **10.6 (9.2)** | 2.2 (0.6) |
| 6 Pos2 | **9.0** | 23.7 | 14.7 | 11.1 | 10.8 | **10.2** | 2.7 |
| 7 Pos1 | **0.9 (0.9)** | 15.6 (15.6) | 4.2 (4.2) | 3.8 (3.8) | 3.4 (3.4) | **3.3 (3.3)** | 0.1 (0.1) |
| 7 Pos2 | **2.4** | 11.2 | 5.0 | 4.9 | 4.6 | **4.3** | 0.7 |
| 8 Pos1 | **4.4 (1.1)** | 44.2 (25.5) | 15.1 (15.4) | 1.0 (1.0) | 0.7 (0.7) | **0.6 (0.6)** | 0 (0) |
| 8 Pos2 | **3.6** | 48.6 | 20.6 | 17.4 | 157 | **11.7** | 0 |
| 9 Pos1 | **20.3 (20.3)** | 38.6 (38.6) | 26.3 (26.3) | 25.2 (25.2) | 24.4 (24.4) | **23.4 (23.4)** | 5.6 (5.6) |
| 9 Pos2 | **22.3** | 40.4 | 28.7 | 26.8 | 25.2 | **23.5** | 3.2 |
| 10Pos1 | **8.4 (8.4)** | 29.8 (29.8) | 20.5 (20.5) | 18.4 (18.4) | 15.1 (15.1) | **13.0 (13.0)** | 0.3 (0.3) |
| 10Pos2 | **9.5** | 31.8 | 15.7 | 13.7 | 12.9 | **12.3** | 1.7 |
| 11Pos1 | **189.8(189.8)** | 371.6(371.6) | 49.2(217.0) | 194.1(194.1) | 184.9(184.9) | **180.2(180.2)** | 8.8 (8.8) |
| 11Pos2 | **181.2** | 352.0 | 229.2 | 194.8 | 187.6 | **181.4** | 38.4 |
| 12Pos1 | **0.1 (0.1)** | 9.9 (9.9) | 2.5 (2.5) | 1.0 (1.0) | 0.9 (0.9) | **0.7 (0.7)** | 0 (0) |
| 12Pos2 | **0.2** | 9.3 | 2.2 | 2.1 | 1.7 | **1.5** | 0 |
| 13Pos1 | **8.1 (8.1)** | 26.1 (26.1) | 16.4 (16.4) | 12.9 (12.9) | 12.5 (12.5) | **11.9 (11.9)** | 0.3 (0.3) |
| 13Pos2 | **8.8** | 27.3 | 12.9 | 12.4 | 11.9 | **11.3** | 0 |
| 14Pos1 | **1.5 (1.5)** | 13.4 (13.4) | 5.8 (5.8) | 5.7 (5.7) | 4.9 (4.9) | **3.6 (3.6)** | 0.1 (0.1) |
| 14Pos2 | **1.5** | 17.0 | 6.3 | 5.7 | 5.3 | **4.8** | 0 |
| 15Pos1 | **37.2 (37.2)** | 91.5 (91.5) | 60.2 (60.2) | 55.0 (55.0) | 53.0 (53.0) | **50.6 (50.6)** | 9.5 (9.5) |
| 15Pos2 | **33.2** | 82.6 | 60.8 | 59.1 | 56.6 | **55.0** | 4.9 |
| 16Pos1 | **62.0 (62.0)** | 153.2(153.2) | 106.1(106.1) | 98.1(98.1) | 95.3 (95.3) | **85.0(85.0)** | 11.4(11.4) |
| 16Pos2 | **60.0** | 147.9 | 109.1 | 100.0 | 97.4 | **92.9** | 16.5 |
| 17Pos1 | **143.2(143.2)** | 192.5(192.5) | 158.3(158.3) | 149.2(149.2) | 146.1(146.1) | **142.1(142.1)** | 31.2(31.2) |
| 17Pos2 | **139.9** | 195.3 | 156.8 | 150.0 | 142.9 | **140.2** | 23.7 |
| 18Pos1 | **256.6(256.6)** | 457.1(457.1) | 313.8(313.8) | 293.2(293.2) | 285.5(285.5) | **270.8(270.8)** | 47.1(47.1) |
| 18Pos2 | **245.2** | 405.8 | 308.9 | 293.9 | 285.1 | **266.8** | 52.0 |
| 19Pos1 | **25.5 (25.5)** | 46.7 (46.7) | 34.8 (34.8) | 33.5 (33.5) | 32.7 (32.7) | **31.8 (31.8)** | 5.8 (5.8) |
| 19Pos2 | **26.6** | 52.7 | 37.7 | 36.4 | 4.6 | **31.2** | 3.6 |
| 20Pos1 | **6.8 (7.3)** | 34.0 (34.0) | 12.1 (17.6) | 9.6 (13.6) | 8.7 (12.5) | **7.3 (12.0)** | 0 (0.9) |
| 20Pos2 | **7.8** | 34.1 | 12.8 | 11.0 | 9.9 | **9.4** | 0.3 |
| 21Pos1 | **33.1 (33.1)** | 75.7 (75.7) | 47.1 (47.1) | 43.9 (43.9) | 42.5 (42.5) | **40.2 (40.2)** | 8.3 (8.3) |
| 21Pos2 | **34.5** | 67.4 | 45.1 | 39.5 | 36.6 | **35.4** | 13.2 |
| 22Pos1 | **7.0 (7.0)** | 371.6(371.6) | 217.0(217.0) | 11.2 (11.2) | 9.8 (9.8) | **9.5 (9.5)** | 0 (0) |
| 22Pos2 | **6.4** | 652.0 | 229.0 | 12.2 | 12.0 | **11.7** | 0.1 |
| 23Pos1 | **6.8 (6.8)** | 34.0 (34.0) | 12.1 (12.1) | 9.6 (9.6) | 8.7 (8.7) | **7.3 (7.3)** | 0 (0) |
| 23Pos2 | **7.8** | 34.1 | 12.8 | 11.0 | 9.9 | **9.4** | 0.3 |
| 24Pos1 | **33.7 (33.7)** | 65.1 (65.1) | 52.3 (52.3) | 50.8 (50.8) | 50.0 (50.0) | **49.4 (49.4)** | 11.8(11.8) |
| 24Pos2 | **34.0** | 91.4 | 51.9 | 48.9 | 47.1 | **46.1** | 7.5 |
| 25Pos1 | **31.7 (31.7)** | 85.5 (85.5) | 53.2 (53.2) | 44.1 (44.1) | 42.6 (42.6) | **41.5 (41.5)** | 5.0 (5.0) |
| 25Pos2 | **31.7** | 68.9 | 53.8 | 47.8 | 45.7 | **43.1** | 5.9 |
| 26Pos1 | **12.6 (12.6)** | 609.5(609.5) | 20.2 (20.2) | 19.1 (19.1) | 18.4 (18.4) | **14.8 (14.8)** | 0.5 (0.5) |
| 26Pos2 | **10.8** | 622.1 | 20.0 | 16.6 | 16.0 | **15.6** | 0.3 |

Attachment, AS in all lesions. Position 1 measured twice (second measurement within ())
